# 

**Published:** 1880-03

**Authors:** 


LUCIEN HOWE, M. D.

### CONTENTS:

PAGE.

ORIGINAL COMMUNICATIONS:

The Relation of Membranous Croup to Diphtheria, by J. O. Roe, M. D., . . 325

CLINICAL REPORTS:

Extra-Uterine Pregnancy; Death and Removal of Fœtus by Prof. J. P. White, M. D., reported by William D. Granger, M. D., . . . . . . . . . 341

Strictura Ilei, Laparo-enterotomy, reported by Herman Mynter, M. D., . . . . 347

A Fracture Case. Service of Dr. C. C. F. Gay, reported by Dr. F. Peterson, . . 351

TRANSLATIONS:

The Use of Iodide of Potassium and Calomel in Diseases of the Eye. Translated from the German by Lucien Howe, M.D., 352

SELECTIONS: PAGE.

Bromide of Ethyl—the New Anæsthetic, . 353

Peculiarities of Ringworm and its Treatment, . . . . . . . . . 355

Treatment of Asthma by Potassium Iodide, . 356

Treatment of Whooping Cough by Atropia, by Arthur Wiglesworth, L. R. C. P., &c., 357

The Treatment of Phthisical Cough, . . 360

Balsam of Peru in Pruritis, . . . . . 361

SOCIETY REPORTS:

Buffalo Medical Association, . . . . . 361

EDITORIAL:

The University of Buffalo, . . . . . 363

Vital Statistics of Buffalo, . . . . . 365

The Thirty-third Commencement of the Buffalo Medical College, . . . . . 366

Maltine, . . . . . . . . . 368

REVIEWS, . . . . . . . 369

